# S126. GOOD OUTCOME IN INDIVIDUALS AT ULTRA-HIGH RISK (UHR) OF DEVELOPING PSYCHOSIS: A DELPHI STUDY

**DOI:** 10.1093/schbul/sby018.913

**Published:** 2018-04-01

**Authors:** Natalia Petros, Andrea Mechelli, Paolo Fusar-Poli, Sandra Vieira, Emma Rowland, Philip McGuire

**Affiliations:** 1 Institute of Psychiatry, Psychology and Neuroscience, King’s College London; 2 Institute of Psychiatry Psychology and Neuroscience, King’s College London, University of Pavia; 3 King’s College London

## Abstract

**Background:**

Long-term outcomes for individuals at risk of developing psychosis are heterogeneous; some develop a psychotic disorder, others continue to experience attenuated psychotic symptoms (APS) and some experience clinical remission and functional recovery. Existing UHR literature is primarily vulnerability- and disease-focused. In recent years, there has been a gradual shift in research to focus on more favourable outcomes, yet despite positive findings, very few UHR studies have directly investigated or even reported good outcomes in this population. Perhaps one major obstacle for this research is the lack of a sound definition of what constitutes a ‘good’ outcome for UHR individuals. The current study uses the Delphi method to systematically reach a consensus definition, amongst UHR clinical and research experts, of good outcome in this clinical population. To our knowledge, this is the first UHR-focused study to utilize the Delphi method.

**Methods:**

A three-round online Delphi study was conducted and n=135 UHR-expert clinicians and researchers drawn from multiple continents were invited to take part. In Round 1, participants were asked to: i) select from a list of items those which they considered most important to the definition of good outcome in UHR and ii) make suggestions on ways in which good outcome could be determined in a standardised way. In Round 2, participants were asked rate the importance of each item to good outcome in UHR individuals, on a 5-point Likert scale. According to the proportion of participants who rated the items as ‘essential’ or ‘important’, items were: i) accepted as part of the consensus and included as a standard if rated by ≥80% or more of the group, ii) re-introduced in the third round and participants were given the opportunity to re-rate them if rated by between 50–79% of the group or iii) excluded if rated by less than 50% of participants.

**Results:**

Forty-six (34.1%) participants responded to the first round of the Delphi process, 39 (84.7% retention rate) responded to Round 2, and 30 (76.9% retention rate) to Round 3. Of the 46 UHR-experts, 20 were psychiatrists, 17 were psychologists, 8 were researchers/lecturers and 1 was a social worker. Fifteen items were endorsed by ≥80% of the expert-participants as ‘essential’ or ‘important’ for defining UHR-specific good outcome at one-year follow-up. Items fell into one of the following categories: functioning, symptoms, other clinically relevant factors or personal wellbeing. ‘Daily functional capacity’ and and ‘self-reported improvement in mental health’ were rated as ‘essential’ to defining good outcome for an UHR individual at one-year, by 100% of the Delphi sample. A reduction in the distress associated with APS was deemed ‘important’ by 92.1% of the sample, more so than the complete remission of APS. Many similar items were rated with the same level of endorsement for the question on outcome at five-year follow-up. Twenty-one protective factors reached ≥80% endorsement for being essential or important for good outcome in UHR individuals and fell into at least one of the following categories: community support; mental health services support; cognitive factors; personal wellbeing; social network/support; substance use/abuse; daily living factors; and premorbid factors.

**Discussion:**

This three-phase Delphi study achieved consensus on the core features of good outcome at one-year and five-years in the UHR population. The items that form this definition could be used in future research and clinically, to evaluate treatment and outcome of UHR individuals. They can also be of value to the development of intervention frameworks. Further studies involving other stakeholder groups, particularly individuals considered to be at risk of developing psychosis, are needed.

